# *DELE1* haploinsufficiency causes resistance to mitochondrial stress-induced apoptosis in monosomy 5/del(5q) AML

**DOI:** 10.1038/s41375-023-02107-4

**Published:** 2023-12-15

**Authors:** Jean-François Spinella, Jalila Chagraoui, Céline Moison, Vincent P. Lavallée, Isabel Boivin, Deanne Gracias, Sylvie Lavallée, Guillaume Richard Carpentier, François Beliveau, Josée Hébert, Guy Sauvageau

**Affiliations:** 1 Institute for Research in Immunology and Cancer, Université de Montréal, Montréal, QC Canada; 2 The Leucegene Project, Université de Montréal, Montréal, QC Canada; 3 Sainte-Justine Research Center, Centre Hospitalier Universitaire Sainte-Justine, Montréal, QC Canada; 4 Department of Pediatrics, Université de Montréal, Montréal, QC Canada; 5 Leukemia Cell Bank of Quebec, Maisonneuve-Rosemont Hospital, Montréal, QC Canada; 6 Princess Margaret Cancer Centre, University Health Network, Toronto, ON Canada; 7 Department of Medicine, Division of Medical Oncology and Hematology, Temerty Faculty of Medicine, University of Toronto, Toronto, ON Canada; 8 Division of Hematology, Maisonneuve-Rosemont Hospital, Montréal, QC Canada; 9 Department of Medicine, Faculty of Medicine, Université de Montréal, Montréal, QC Canada

**Keywords:** Acute myeloid leukaemia, Cancer genomics

## Abstract

Monosomy 5 and deletions of the chromosome 5q (−5/del(5q)) are recurrent events in de novo adult acute myeloid leukemia (AML), reaching up to 40% of cases in secondary AML. These chromosome anomalies are associated with *TP53* mutations and with very poor prognosis. Using the large Leucegene genomic and transcriptomic dataset composed of 48 −5/del(5q) patient specimens and 367 control AML, we identified *DELE1* – located in the common deleted region – as the most consistently downregulated gene in these leukemias. *DELE1* encodes a mitochondrial protein recently characterized as the relay of mitochondrial stress to the cytosol through a newly defined OMA1-DELE1-HRI pathway which ultimately leads to the activation of ATF4, the master transcription factor of the integrated stress response. Here, we showed that the partial loss of *DELE1* expression observed in −5/del(5q) patients was sufficient to significantly reduce the sensitivity to mitochondrial stress in AML cells. Overall, our results suggest that *DELE1* haploinsufficiency could represent a new driver mechanism in −5/del(5q) AML.

## Introduction

Monosomy 5 and deletions of the chromosome 5q (−5/del(5q)) are recurrent chromosomal abnormalities detected in about 5% of de novo adult acute myeloid leukemia (AML) and up to 40% in secondary AML [1]. −5/del(5q) alterations are enriched in complex karyotype (CK) AML and are associated with a high incidence of *TP53* mutations [2]. In contrast to myelodysplastic neoplasms (MDS), presenting low blasts and isolated 5q deletion (MDS-5q) associated with a good prognosis [3, 4], −5/del(5q) AML show low rates of complete remission, a high relapse occurrence and an adverse genetic risk in the 2022 European LeukemiaNet recommendations for AML [5].

Even though several candidate haploinsufficient genes (e.g. *EGR1, APC, CTNNA1, CDC25C, CSNK1A1*) located in or out of the common deleted region (CDR) have been proposed [6–9], the contributing genetic events leading to this poor outcome remain unclear.

Here, using whole genome sequencing (WGS) combined with comparative transcriptomic approaches on the Leucegene cohort (https://data.leucegene.iric.ca/), we identified *DELE1* (Death Ligand Signal Enhancer) – located in the CDR and coding for a protein associated with the inner mitochondrial membrane – as the most consistently under-expressed gene in −5/del(5q) AML, over other currently known culprits. We show that AML cells presenting a partial loss of *DELE1* expression fail to activate the newly defined OMA1-DELE1-HRI pathway [10, 11] and downstream ATF4 signaling, resulting in a resistance to mitochondrial stress-induced apoptosis, and suggesting *DELE1* as a new haploinsufficient driver gene that may contribute to −5/del(5q) AML phenotype.

## Material and methods

### Primary AML specimens

The Leucegene project is an initiative approved by the Research Ethics Boards of Université de Montréal and Maisonneuve-Rosemont Hospital. Leucegene AML samples (−5/del(5q): *n* = 48; control AML: *n* = 367, of which 27 were CK AML with no deletion of chromosome 5) were collected between 2001 and 2015 and characterized by the Banque de cellules leucémiques du Québec (BCLQ) after obtaining an institutional Research Ethics Board–approved protocol with informed consent according to the Declaration of Helsinki. The Quebec Leukemia Cell Bank is a biobank certified by the Canadian Tissue Repository Network. Detailed information on the cohort was previously published (see https://leucegene.ca/ and Moison et al. [12]). Cytogenetic aberrations and composite karyotypes were described according to the International System for Human Cytogenomic Nomenclature 2020 guidelines [13]. Complex karyotype (CK) was defined as having 3 or more chromosomal abnormalities in the absence of one of the WHO-designated recurring alterations: t(8;21), inv(16) or t(16;16), t(9;11), t(v;11)(v;q23.3), t(6;9), inv(3) or t(3;3) and t(9;22) [14].

### Low-pass (low coverage) whole genome sequencing and data analysis

Tumor and normal (when available) gDNAs were sequenced on NovaSeq6000 S4 (paired-end 150 bp). Alignment to GRCh38 was done using the BWA aligner (v0.7.12) [15], PCR duplicates were marked using Picard [16] and a GATK (v4.1.0) [17] base quality score recalibration was applied. A mean depth coverage ~5X was reached for each sample. Identification of regions of genomic gains and losses was done using FREE-Copy number caller (FREEC, v11.5) [18]. The optimization of algorithm parameters (breakPointThreshold = 1.4, window = 100,000, step = 13,000, readCountThreshold = 20, contaminationAdjustment = “TRUE”, minMappabilityPerWindow = 0.95, breakPointType = 4, minCNAlength = 1) was conducted using known alterations as reference. The concatenation of adjacent CNVs was done using the *merge* option of Bedtools (v2.25.0) [19].

### RNA sequencing and data analysis

Leucegene RNAseq libraries were constructed according to TruSeq Protocols (Illumina) and sequencing was performed using an Illumina HiSeq 2000/4000 instrument. Pseudo-alignment and quantification of transcripts were done using Kallisto (v0.46.0) [20] with hg38 transcriptome as reference. The Tximport R package [21] was used to obtain a per gene quantification. The limma package and its Voom method [22] was used to conduct differential expression analysis. Point mutations were identified from RNAseq data as previously described [12]. MCC (Matthews Correlation Coefficient) values reported in the Table S4 were calculated using EPCY [https://github.com/iric-soft/epcy, v0.0.1] on expression data (TPM), comparing −5/del(5q) AML to other AML from the Leucegene cohort.

### Cell line culture

OCI-AML1 and OCI-AML5 cell lines were purchased from ATCC and maintained in alpha-MEM, 10% heat-inactivated FBS, and 10 ng/ml GM-CSF (Shenandoah). HL60 and K562 were cultured in RPMI1640 and 10% heat-inactivated FBS. HEK293, HeLa and HCT116 cell lines were cultured in DMEM supplemented with 10% heat-inactivated FBS. All cell lines were grown in humidified incubators at 37^o^C and 5% CO2.

### Human cord blood cell collection and processing

This study was approved by the Research Ethics Boards of Université de Montréal and Charles LeMoyne Hospital (Greenfield Park, QC, Canada). All umbilical cord blood units were collected from consenting mothers at the Charles LeMoyne Hospital (Greenfield Park, QC, Canada). Human CD34+ cord blood (CB) cells were isolated using The EasySep™ positive selection kit (StemCell Technologies Cat #18056). CB cells were cultured in expansion media consisting of StemSpan SFEM (StemCell Technologies) supplemented with human 100 ng/ml stem cell factor (SCF, R&D Systems), 100 ng/ml FMS-like tyrosine kinase 3 ligand (FLT3, R&D Systems) and 50 ng/ml thrombopoietin (TPO, R&D Systems).

### Plasmids and gene transfer

Lentiviral vectors carrying shRNAs (shDELE1) were generated by cloning appropriate shRNA sequences as described in (Fellmann et al. [23]) into MNDU vectors comprising miR-E sequences as well as GFP. shRNAs targeting Renilla luciferase were used as control (shLuc). Guide sequences are as follow: shDELE1#1: TTTTGATTTATCTTGTTCCTTT; shDELE1#2: TCTCATAGCAAATTCCAAGGTG. Lentiviral vectors carrying AFTF4 translational reporter (ATF4 reporter followed by mApple fluorescent protein) were purchased from Addgene (#141281). Lentiviruses were produced in HEK-293 cells and AML cell lines or primary CD34+ cells were infected with lentiviruses in media supplemented with 10 ng/mL polybrene for 24 h. Infection efficiency, as determined by the percentage of GFP positive cells, was monitored by flow cytometry using a BD FACSCantoII flow cytometer. When needed, infected cells were sorted using a BD Aria II cell sorter and knockdown efficiency was determined by Q-PCR using standard methods.

### Chemicals

Oligomycin A (Sigma-Aldrich, #75351), Thapsigargin (Sigma-Aldrich, #T9033), CCCP (Sigma-Aldrich, #C2920) and cytarabine (Tocris, #4520) were used as indicated in figures.

### Dose responses and treatments

AML cell lines were plated in 384-well plates, 300 cells per well in 50 μL. Oligomycin was dissolved in DMSO and added to seeded cells in serial dilution (8 dilutions, 1:4, 500 nM down to 0.025 nM). Cell viability was evaluated after 4 days in culture using the CellTiterGlo assay (Promega) according to the manufacturer’s instruction. Percentage of inhibition for dose response curves was calculated as 100 – (100 x (mean luminescence [compound]/mean luminescence [DMSO])), IC50 values are reported in figure. Cord blood cells were seeded in 48-well plates and exposed to 250 or 500 nM of oligomycin for 72 h. Viability and percentage of GFP-positive cells was determined by flow cytometry and compared to DMSO-treated cord blood cells. AML cell lines-engineered to express shRNAs and/or ATF4 reporter were exposed to DMSO or CCCP (5, 10 or 20 uM) for the indicated time.

### Western blot analysis

Total protein extraction was performed in RIPA lysis buffer (20 mM Tris-HCl pH7.4, 150 mM NaCl, 5 mM MgCl2, 5 mM EGTA, 60 mM β-glycerophosphate, 0.1% NP40, 0.1% Triton X-114, 1 mM DTT) supplemented with protease and kinase inhibitors (PMFS Sigma P-7626, Aprotinin Sigma A-1153, Leupeptin Sigma L-2884, Glycerophosphate, Na2VO4, NaF), and quantified by the bicinchoninic acid (BCA) method using BSA for standard curve. Proteins were resolved by SDS-PAGE, transferred onto PVDF membrane, blocked with 5% milk and probed with primary (overnight, 4 C) and secondary (1 h, room temperature) antibodies. Primary antibodies: EIF2 (Cell Signaling Technology 9722), pEIF2 (Cell Signaling Technology 3597), OPA1 (BD Biosciences 612606), DDIT3 (Cell Signaling Technology 2895 S), OMA1 (ProteinTech Group 17116-1-AP) and alpha-tubulin (Cell Signaling Technology 2144 S). Secondary antibodies: anti-mouse HRP (Jackson ImmunoResearch 115-035-146) and anti-rabbit HRP (Jackson ImmunoResearch 111-035-144).

### 7AAD assays

HEK293, HeLa and HCT116 cell lines were transfected with 10 µg DNA (DELE1 (Plasmid #141283) or EGFP (CTRL)) using JetPrime reagent following the manufacturer’s recommended protocol (PolyPlus). Cell death was assessed 48 h after transfection using 7AAD dye (BioLegend).

### Statistical analysis

Statistical analyses of all experiments were done using R. Depending on the dataset, Fisher’s Exact test or Mann–Whitney test were used to determine significance (*p*-value < 0.05).

## Results

**Fig. 1:**
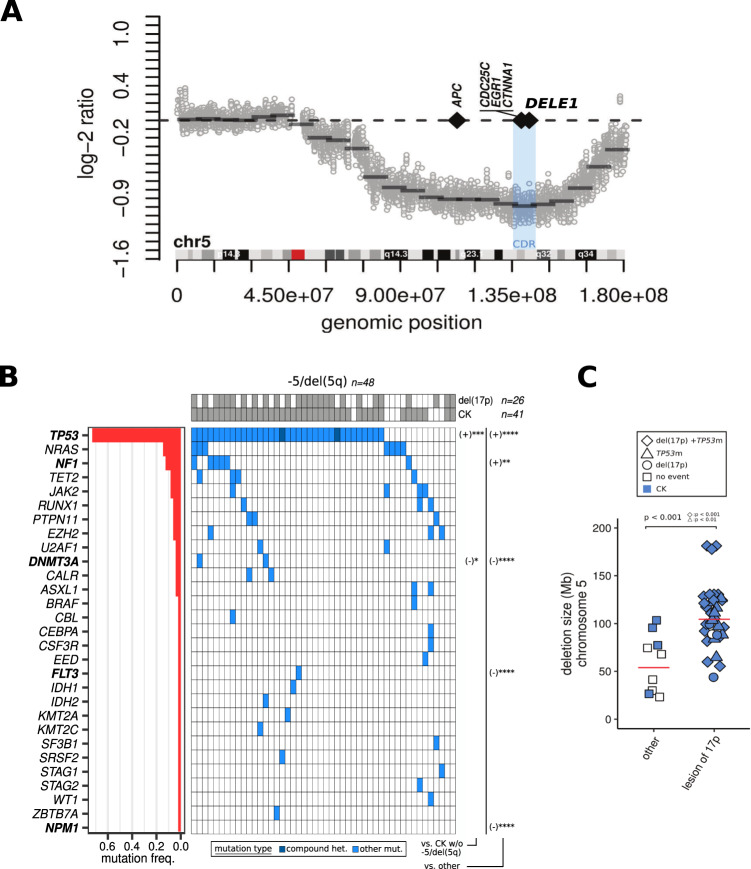
5q common deleted region and mutation landscape of −5/del(5q) AML. **A** Median log-2 copy number ratio (log-2 ratio) calculated for windows of 13 Kb (depicted by gray dots; each dot represents the median value obtained for the whole −5/del(5q) WGS cohort for a specific window) covering chromosome 5 (genomic position and schematic representation of chromosome 5 are indicated along the *x*-axis). Gray and red squares on the schematic representation of chromosome 5 depict cytobands and the centromere, respectively. The horizontal dashed black line marks a log-2 ratio of 0 corresponding to a normal diploid state. Black solid segments represent the median log-2 ratio calculated for overlapping windows of 10 Mb (overlap = 2.5 Mb). The vertical blue zone delimits the CDR (5q31.1 to 5q31.3). The position of genes of interest are indicated by black diamonds. **B** Mutations heatmap of −5/del(5q) AML. Genes (*y*-axis) composing the heatmap were either mutated in one or more −5/del(5q) specimens (*x*-axis). *NPM1* was conserved because its wild-type status was significantly associated with −5/del(5q) AML compared to other AML or to complex karyotype (CK) AML without −5/del(5q). Genes were ordered (from top to bottom) based on their mutation frequencies (indicated by the red bar graph on the left). Specimens were grouped according to their mutation status (from left to right). Specimens presenting a deletion of the chromosome 17p (del(17p)) spanning *TP53* (identified by FISH and/or WGS) or a complex karyotype (CK) are flagged by gray cells at the top of the heatmap. Mutation types (compound heterozygous or other mutations) are depicted by dark and light blue cells in the heatmap, as indicated in the legend at the bottom of the heatmap. Significant associations or anti-associations (depicted by a (+) and a (−), respectively) compared to other AML or to complex karyotype (CK) AML without −5/del(5q) (as indicated at the bottom right of the heatmap) are shown for concerned genes (in bold) at the right of the heatmap. *, **, *** and **** stand for *p* < 0.05, *p* < 0.01, *p* < 0.001 and *p* < 1e-04, respectively. **C** Size of chromosome 5 deletions for patients with or without lesions of chromosome 17p. Median values are indicated by red lines. As indicated by the legend, diamonds depict patients with a combined deletion of the chromosome 17p (del(17p)) and a mutation of *TP53* (*TP53*m), triangles depict *TP53*m without del(17p) and dots depict del(17p) without *TP53*m. Squares represent patients without lesions of the 17p (indicated as “other” on the *x*-axis). Blue symbols represent −5/del(5q) specimens presenting a complex karyotype (CK). *P*-values resulting from the tests comparing each group to the “other” AML are directly indicated on the figure.

Cytogenetics data were used to initially define the −5/del(5q) cohort (*n* = 48 AML with a clonal 5/5q deletion, of which 41 belonged to the CK subgroup, see Table 1 for clinical characteristics) in a cohort composed of a total of 415 AMLs (other specimens being subsequently used as controls, *n* = 367). Complementarily, low-pass WGS data (available for *n* = 42 −5/del(5q) specimens, Methods) allowed us to refine 5q deletion and CDR boundaries. Using these data, we identified 40 patients carrying large deletions of chromosome 5 – from 23.2 Mb to 177.8 Mb – while two cases presented a complete loss of the chromosome (overall median size of 98.2 Mb). The calculation of the median log-2 copy number ratio along chromosome 5 revealed a global minimum value of −0.99 for a region extending from 5q31.1 to 5q31.3 cytobands corresponding to the previously reported CDR [24] (blue shade in Fig. 1A, Fig. S1) which includes known candidate haploinsufficient genes such as *CDC25C*, *EGR1* and *CTNNA1* [6–8].

**Table 1:** Clinical characteristics of −5/del(5q) AML.

	−5/del(5q) (*n* = 48)	other (*n* = 367)	*P*-value
Age	65 (37–87)	57 (17–87)	<1e–3^a^
WBC (x10^9^/L)	11.5 (0.8–321)	35.8 (0.8–447)	<1e–4^a^
Gender			
Female	21 (43.7%)	159 (43.3%)	—
Male	27 (56.2%)	208 (56.7%)	—
Cytogenetic risk			
Adverse	48 (100%)	74 (20.1%)	<1e–4^b^
Intermediate	0 (0%)	230 (62.7%)	—
Favorable	0 (0%)	63 (17.2%)	—
Cytogenetic group			
CK	41 (85.4%)	27 (7.4%)	<1e–4^b^
FAB			
M0	8 (16.7%)	19 (5.2%)	<0.01^b^
M1	7 (14.6%)	109 (29.7%)	<0.05^b^
M2	7 (14.6%)	45 (12.3%)	—
M3	0 (0%)	15 (4.1%)	—
M4	2 (4.2%)	54 (14.7%)	—
M5	1 (2.1%)	65 (17.7%)	—
M6	5 (10.4%)	5 (1.4%)	—
M7	1 (2.1%)	2 (0.5%)	—
NC	17 (35.4%)	53 (14.4%)	—

The “other” cohort is constituted of AML cases from diverse cytogenetic subgroups composing the Leucegene cohort.

*WBC* white blood cell count, *CK* complex karyotype, *FAB* French-American-British.

^a^Mann–Whitney test.

^b^Fisher’s exact test.

### Genetic alterations of *TP53* are associated with larger deletions of chromosome 5

Consistent with previous reports, *TP53* was the most frequently mutated gene in −5/del(5q) patients (37 mutations in 35 specimens) [2] and presented an excess of mutations in the −5/del(5q) cohort when compared to other AML or to CK AML without −5/del(5q) (*p* < 1e–04 and *p* < 0.001, respectively; Fig. 1B and Table S1), suggesting a cooperative effect with the deletion.

As reported in a previous Leucegene study on CK AML [12], more than 80% (30/37) of *TP53* mutations presented a variant allele frequency (VAF) > 0.75 (Fig. S2 and Table S1), reflecting the predominant expression of the mutated allele. Using WGS data, we completed fluorescent in situ hybridization (FISH) experiments targeting *TP53* on chromosome 17p, allowing the identification of deletions including *TP53* in a group of 26 cases enriched for point mutations in the gene (22/26, *p* = 0.06; see del(17p) track in Fig. 1B and Table S1) and explaining the majority of unbalanced expressions; remaining high VAF TP53 mutations without identified 17p deletion probably being due to copy neutral loss of heterozygosity (cnLOH) events which are not detectable from low-pass WGS data. Overall, more than 80% of −5/del(5q) specimens harbored an alteration of *TP53* (mutation and/or deletion, *n* = 39/48; Fig. 1B and Table S1).

Corroborating previous studies [24, 25], lesions of chromosome 17p were significantly associated with larger deletions of chromosome 5 (*p* < 0.001; Fig. 1C), which remained true considering *TP53* mutations only (*p* < 0.01; Fig. 1C). This is in line with the hypothesis that larger lesions involving genomic regions distant from 5q CDR could include genes for which the loss of copy cooperates with alterations of *TP53* [25].

### *DELE1* is the most consistently downregulated gene in −5/del(5q) AML

**Fig. 2:**
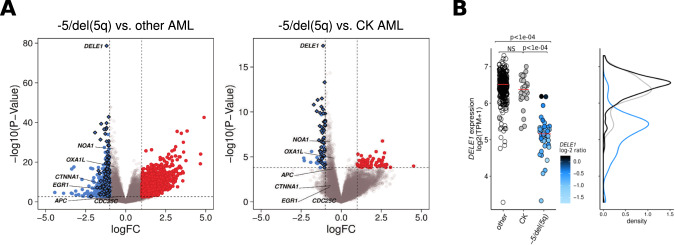
*DELE1* is the most consistently downregulated gene in −5/del(5q) AML. **A** Volcano plot representation of the differential expression analysis conducted on RNAseq data comparing −5/del(5q) AML samples (*n* = 48) versus (left panel) other AML (*n* = 367) or (right panel) complex karyotype (CK) AML without −5/del(5q) (*n* = 27) as control. The horizontal dashed line indicates an adjusted *p*-value of 0.01 and vertical dashed lines indicate log fold change (logFC) of 1 and -1. Red and blue dots correspond to genes significantly over- and under-expressed (logFC > |1|, FDR < 0.01), respectively. Diamonds depict genes significantly downregulated and located on the chromosome 5q. **B** Dotplot representation (left panel) and density curves (right panel) of *DELE1* expression in the −5/del(5q) cohort (*n* = 48, in blue), CK specimens without −5/del(5q) (*n* = 27, in gray) and other AML (*n* = 340, in black). Median values are indicated by red lines on each dotplot. Adjusted *p*-values resulting from the differential expression analysis are directly indicated on the figure. The color code for the −5/del(5q) group is representative of the median log-2 copy number ratio (log-2 ratio) calculated for the genomic region of *DELE1* (window centered on the gene and extended for 25 kb on each side).

Differential expression analysis of −5/del(5q) AML was conducted using other AML from the Leucegene cohort as controls or complex karyotype AML without −5/del(5q) (Fig. 2A, Tables S2 and S3). While a trend existed between expression and the estimated copy number of 5q genes (Pearson’s *r* = 0.35; Fig. S3), previously identified haploinsufficient candidates, such as *CDC25C and CTNNA1*, showed a limited to no drop of expression when compared to other AML (logFC = −0.06 and −0.8, respectively) or to CK AML (logFC = 0.1 and −0.6, respectively; Fig. 2A, Fig. S4A, B). As for *EGR1*, expression levels were highly variable across −5/del(5q) specimens (σ^2^(log2(TPM + 1)) = 1.40) leading to a decrease of its significance (Fig. 2A, Fig. S4C). Overall, the loss of copy of these genes rather showed a case-specific expression footprint with several specimens presenting control-like levels – as evidenced by their MCC (Matthews Correlation Coefficient) values: 0.22, 0.04, and 0.33 for *EGR1*, *CDC25C* and *CTNNA1*, respectively (Table S4, Supplemental Methods) – despite low copy number ratios (Fig. S4).

On the other hand, our analysis identified *DELE1*, located in the CDR downstream of the other candidates (Fig. 1A), as the most consistently down-expressed gene (logFC = −1.2, FDR = 7.3e–75, σ^2^(log2(TPM + 1)) = 0.32 and top 1 MCC value = 0.77; Fig. 2A, B, Table S4), making it an interesting new candidate for haploinsufficiency. Importantly, *DELE1* was also the most significantly downregulated gene when CK AML were used as controls (logFC = −1.1, FDR = 1.2e–13; Fig. 2A).

### *DELE1* down-expression reduces AML cells sensitivity to mitochondrial stress

**Fig. 3:**
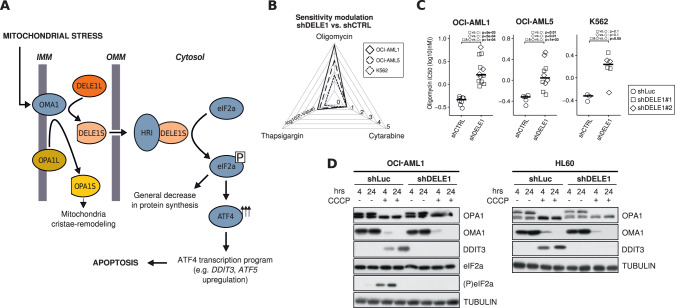
*DELE1* down-expression modulates OMA1–DELE1–HRI pathway. **A** Schematic representation of the OMA1-DELE1-HRI pathway recently defined by Fessler et al. [10] and Guo et al. [11]. *S* and *L* suffixes indicate short and long forms of OPA1 and DELE1. IMM: inner mitochondrial membrane, OMM: outer mitochondrial membrane. **B** Radar plot summary representation of the significance of sensitivity reduction to oligomycin, cytarabine and thapsigargin (-log10(*P*-values), Mann-Whitney tests on IC50 values) for OCI-AML-1, OCI-AML-5 and K562 cells (see legend) expressing shRNA vectors targeting *DELE1* (shDELE1#1 and shDELE1#2) or the luciferase as control (shLuc). **C** Oligomycin IC50 values (log10 of concentrations in nM of the compound that inhibited cell growth by 50%) for OCI-AML-1, OCI-AML-5 and K562 cells expressing shDELE1 (shDELE1#1 and shDELE1#2) or shLuc. *P*-values resulting from Mann-Whitney tests comparing shDELE1#1 and/or shDELE1#2 vs. shLuc conditions are directly indicated on the figure. **D** Western blot analysis of total proteins extracted from OCI-AML1 (left panel) and HL60 cells (right panel) expressing shDELE1#1 or shLuc (control) and exposed to DMSO or CCCP (20 µM) for 4 and 24 h (hrs). Representative blot showing OPA1, OMA1, DDIT3, eIF2a, (P)eIF2a and TUBULIN as loading control.

Poorly characterized until recently, only a sparse literature is available for DELE1 which was originally described as mediating the death receptor-induced apoptosis [26]. In 2020, two major studies reported a new role for DELE1 as a relay of mitochondrial stress to the cytosol [10, 11] (Fig. 3A). Once cleaved by OMA1 (located on the inner mitochondrial membrane), the short form of DELE1 (DELE1S) accumulates in the cytosol and binds HRI (EIF2AK1), leading to its activation and inducing eIF2a phosphorylation, which promotes the transfer of the integrated stress response (ISR) master transcriptional regulator ATF4 to the nucleus. This ultimately leads to the upregulation of pro-apoptotic proteins such as DDIT3 (CHOP), while slowing down the general protein synthesis process, including the production of anti-apoptotic proteins such as MCL1.

In this context, to determine if a partial loss of *DELE1* expression could modulate the response to mitochondrial stress in AML, we tested the sensitivity of three knockdown leukemia cell lines – virtually presenting the expression drop observed in −5/del(5q) specimens (shRNA-mediated *DELE1*-KD OCI-AML1, OCI-AML5 and K562, Fig. S5) – to three compounds: oligomycin, (inhibitor of ATP synthase used by Guo et al. to trigger the ISR in their recent DELE1 characterization [11]), cytarabine (inhibitor of DNA polymerase activity widely used as chemotherapy drug in AML), thapsigargin (inhibitor of the sarco/endoplasmic reticulum Ca^2+^ ATPase), and showed that three out of three cell lines presented a specific and significant resistance to oligomycin-induced apoptosis (Fig. 3B, C, Fig. S6), indeed suggesting a modulation of the OMA1–DELE1–HRI pathway.

Despite the lack of effective antibodies targeting DELE1 (as already reported by Guo et al. [11]), immunoblotting quantification of different factors of the pathway in OCI-AML1 (*TP53*-WT) and HL60 (*TP53*-null) AML cells upon 4 and 24 h CCCP exposure – a mitochondrial ionophore used as an alternative ISR-inducing drug by Fessler et al. for DELE1 characterization [10] – showed that a downregulation of *DELE1* gene expression was sufficient to prevent OMA1–DELE1–HRI pathway induction in both cell lines in a *TP53*-independent manner (Fig. 3D, Fig. S5). In line with previously published assays [26], the transient overexpression of *DELE1* impaired survival in all tested cell lines (Fig. S7).

**Fig. 4:**
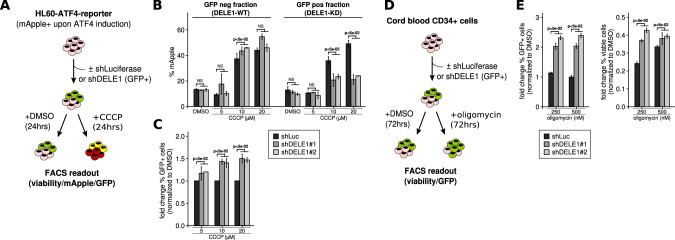
*DELE1* down-expression reduces the sensitivity to mitochondrial stress. **A** Schematic representation of the ATF4 reporter assay in HL60 cells (quantification using flow cytometry). **B** ATF4 induction (% mApple) in the GFP-negative and -positive fractions of HL60 cells expressing shLuc (control), shDELE1 (shDELE1#1 and shDELE1#2) and exposed to DMSO or 5, 10 and 20 µM of CCCP for 24 h (*n* = 3 per shRNA, mean value + standard deviation). *P*-values resulting from Mann-Whitney tests comparing shDELE1#1 and/or shDELE1#2 vs. shLuc conditions are directly indicated on the figure. **C** GFP enrichment in HL60 live cells (GFP ratio, normalized to DMSO) exposed to DMSO or 5, 10 and 20 µM of CCCP for 24 h (*n* = 3 per shRNA, mean value + standard deviation). *P*-values resulting from Mann-Whitney tests comparing shDELE1#1 and/or shDELE1#2 vs. shLuc conditions are directly indicated on the figure. **D** Schematic representation of the GFP-coupled shDELE1 monitoring assay in cord blood CD34+ cells. **E** Enrichment in GFP-positive cord blood cells (fold change of % GFP+ cells normalized to DMSO, left panel) and in viable cells (fold change of live cells normalized to DMSO, right panel) expressing shDELE1 (shDELE1#1 and shDELE1#2) or shLuc (control) exposed to DMSO or 250 and 500 nM of oligomycin for 72 h (*n* = 3 per shRNA, mean value + standard deviation). *P*-values resulting from Mann-Whitney tests comparing shDELE1#1 and/or shDELE1#2 vs. shLuc conditions are directly indicated on the figure.

To specifically monitor ATF4 induction in response to mitochondrial stress in *DELE1*-knockdown cells, we conducted an ATF4-reporter assay in HL60 cells expressing a GFP-coupled-shRNA against *DELE1* and challenged them with CCCP (Fig. 4A). While increasing ATF4 induction levels matching increasing doses of CCCP exposure were observed in GFP negative cells (*DELE1*-WT) and controls, cells expressing the shRNA (GFP+ cells, *DELE1*-KD) showed a limited activation of ATF4 (Fig. 4B, Fig. S8) coupled with increasing proportions of live cells (Fig. 4C), even at the higher dose of CCPP (20 µM), confirming the reduced ability of *DELE1*-KD cells to induce OMA1–DELE1–HRI pathway and suggesting a protective role of *DELE1* downregulation during mitochondrial stress.

Accordingly, *DELE1* shRNA-mediated downregulation in human cord blood cells (Fig. 4D**)** also significantly reduced the sensitivity to oligomycin-mediated mitochondrial stress (Fig. 4E). The observed enrichment in GFP + *DELE1*-KD cord blood cells confirmed the protective effect of *DELE1* downexpression.

## Discussion

In this study, we characterized 415 primary AML specimens of the Leucegene collection by whole genome and transcriptome sequencing, of which 48 presented deletions corresponding to −5/del(5q) AML.

To date, several candidate haploinsufficient genes (e.g. *EGR1, APC, CTNNA1, CDC25C, CSNK1A1*) located in or out of 5q CDR have been studied [6–9]. Modification of *CTNNA1* expression was shown to modulate apoptosis and proliferation in cell lines [7], while *CDC25C* was reported as recurrently mutated in familial platelet disorders with predisposition to acute myelogenous leukemia (FPD/AML) [27] and its dosage demonstrated as influencing the sensitivity to Lenalidomide in del(5q) MDS [28]. More recently, *CSNK1A1* haploinsufficiency was shown to give an advantage to hematopoietic stem cells when compared to haploinsufficient *APC* or *EGR1* in a chronic inflammatory context [9].

Furthermore, given the size of the CDR carrying multiple ORFs, a phenotype depending on several contributing genes cannot be ruled out and few models involving combined alterations of two candidates, such as *APC* and *EGR1* in a *TP53*-null context, have been successfully tested in vivo [6]. While this work confirmed that a cooperative effect of haploinsufficiencies could be at play in −5/del(5q) AML, the combination of contributing events leading to the poor outcome associated with −5/del(5q) AML remains unclear.

While several putative haploinsufficient candidates also located in the CDR have been disregarded – possibly because of a lack of existing characterization for some of these genes – in our data none of the previously reported 5q genes fully matched criteria to be considered as “haploinsufficient candidate”, for they either showed a limited to no drop of expression in −5/del(5q) patients or highly variable expression levels across specimens. However, our approach identified *DELE1* – also located on chromosome 5q but unreported yet in this context – as the most significantly under-expressed gene in Leucegene −5/del(5q) AML.

In 2010, Harada et al. showed that DELE1 binds to the GTP-binding protein DAP3, known to play the role of adapter between TRAIL receptors and FADD, and demonstrated that a variation of *DELE1* expression modulates apoptosis [26]. More recently, DELE1 has been demonstrated as the missing link between mitochondrial stress and ATF4 induction [10, 11, 29] of a mito-nuclear retrograde response, acting via a newly defined OMA1-DELE1-HRI pathway, and ultimately leading to the BAX/BAK-dependent release of cytochrome C and to caspase activation.

Here, we showed that only a partial loss of expression of *DELE1* – mimicking the decreased expression level identified in −5/del(5q) AML – was sufficient to provoke a protective effect from ISR. This study, combined with the growing importance of DELE1 mitochondrial stress relaying function, strongly suggests that its haploinsufficiency should be considered as a new driver candidate participating in −5/del(5q) AML phenotype.

Of note, despite significant enrichment of *TP53* loss of function in −5/del(5q) AML, our experimental data failed to identify a cooperative association between *DELE1* haploinsufficiency and *TP53* alterations. Additionally, considering the association between *TP53* mutations and Venetoclax resistance [30], and given that ATF4 mediates the transactivation of the MCL-1 antagonist NOXA [31], we checked the impact of *DELE1* expression (+/- *TP53* mutations) on Venetoclax response using both Leucegene and BEAT AML data [32], but again did not identify any collaborative effect (Figs. S9 and S10).

Nonetheless, as advocated by recent studies on *DELE1* characterization [10, 11, 29], the influence of its pathways on chemotherapeutic treatments of other tumor types [33], and given the advantage of cells able to cope with mitochondrial perturbation through modification of OMA1-DELE1-HRI signaling, a modulation of this pathway could represent an attractive therapeutic avenue worthing further investigations, especially in the −5/del(5q) AML context which represents a major challenge in terms of clinical management.

### Supplementary information

TableS1

TableS2

TableS3

TableS4

Supplemental figures

## Data Availability

Sequencing data used in this manuscript are available at: https://www.ncbi.nlm.nih.gov/geo/query/acc.cgi?acc=GSE67040.

## Code availability

Codes are available upon request.
