# Supplementary material for: DELE1 haploinsufficiency causes resistance to mitochondrial stress-induced apoptosis in monosomy 5/del(5q) AML
Source: Leukemia. 2023 Dec 15;38(3):530–7. doi: 10.1038/s41375-023-02107-4 (PMC10912023; doi:10.1038/s41375-023-02107-4)
Supplement: Supplementary file 5 — Supplemental figures [file 41375_2023_2107_MOESM5_ESM.pdf]

DELE1 haploinsufficiency alters OMA1-DELE1-HRI signaling and causes resistance to mitochondrial stress-induced apoptosis in -5/del(5q) acute myeloid leukemia

Jean-François Spinella[1], Jalila Chagraoui[1], Céline Moison[1], Vincent P. Lavallée[1], Isabel Boivin[1], Deanne Gracias[1], Sylvie Lavallée[2], Guillaume Richard Carpentier[3], François Beliveau[2], Josée Hébert[1,2,4,5 †], Guy Sauvageau[1,2,4,5 †]

Supplemental figures

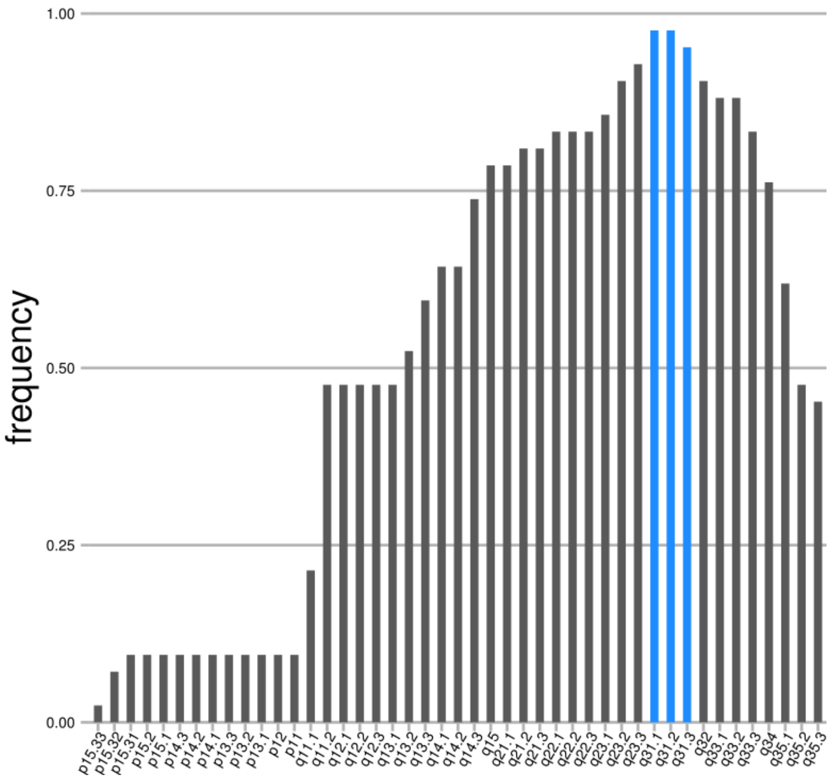

Figure S1. Proportion of deletion events overlapping the different cytobands of chromosome 5 (x-axis) in the Leucegene -5/del(5q) cohort with available WGS data (n = 42). Blue bars correspond to values calculated for cytobands overlapped by the common deleted region (CDR).

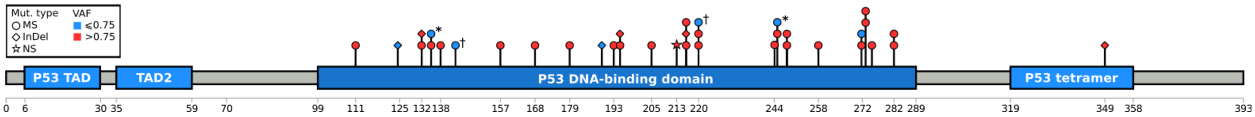

Figure S2. Schematic representation of the *TP53* gene and identified mutations. Dots, diamonds and stars depict missense (MS), frameshift insertions or deletions (InDel) and nonsense (NS) mutations, respectively. Blue and red symbols correspond to VAF (variant allele frequency) ⩽ 0.75 and > 0.75, respectively. Symbols (* and †) indicate compound heterozygous mutations.

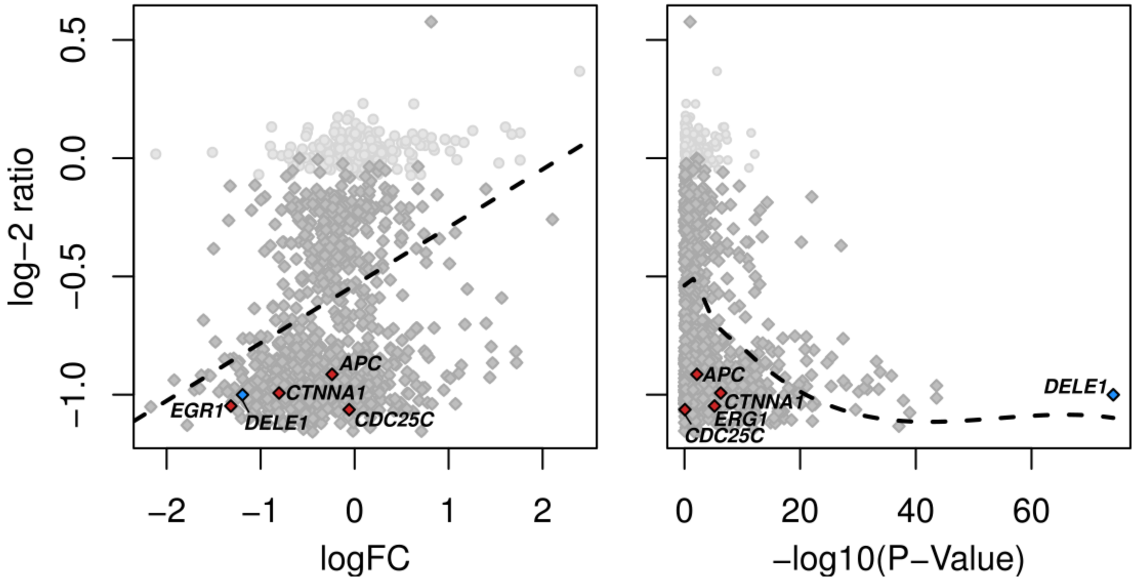

Figure S3. Comparison between the median log-2 copy number ratio (log-2 ratio) calculated for each gene located on chromosome 5 (windows centered on the gene and extended for 25kb on each side) and their log fold change (logFC, left panel) or the adjusted P-Value (right panel) obtained from the differential expression analysis. Light grey dots and dark grey diamonds depict genes located on the small and long arm of chromosome 5, respectively. Dashed lines result from a least squares regression (left panel) and a LOESS regression (right panel). *DELE1* and other 5q candidate genes are indicated by blue and red diamonds, respectively.

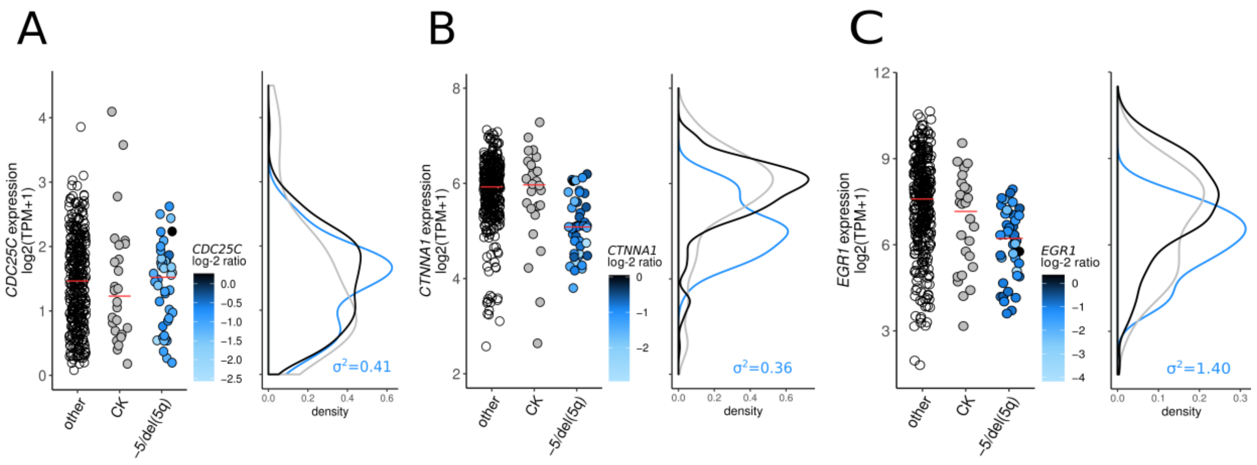

Figure S4. Dotplot representation of (**A**) *CDC25C,* (**B**) *CTNNA1* and (**C**) *EGR1* expression in the -5/del(5q) cohort (n = 48, in blue), CK specimens without -5/del(5q) (n = 27, in grey) and other AML (n = 340, in black). Median values are indicated by red lines on each dotplot. The color code for the -5/del(5q) group is representative of the median log-2 copy number ratio (log-2 ratio) calculated for the genomic region of each gene (window centered on the gene and extended for 25kb on each side).

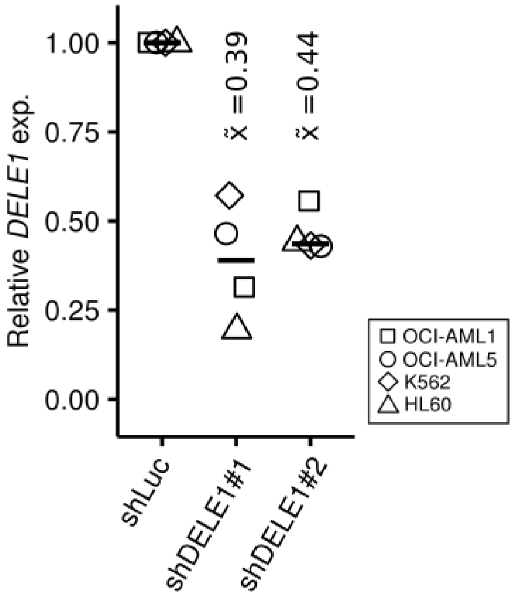

Figure S5. shRNA DELE1 qPCR results in OCI-AML1, OCI-AML5, HL60 and K562. *DELE1* relative expression (ΔΔCT method using endogenous *HPRT* as reference) in cells infected with shRNA vectors targeting *DELE1* (shDELE1#1 and shDELE1#2) or the luciferase as control (shLuc). Median values (x̃) are indicated by black lines on the dotplot.

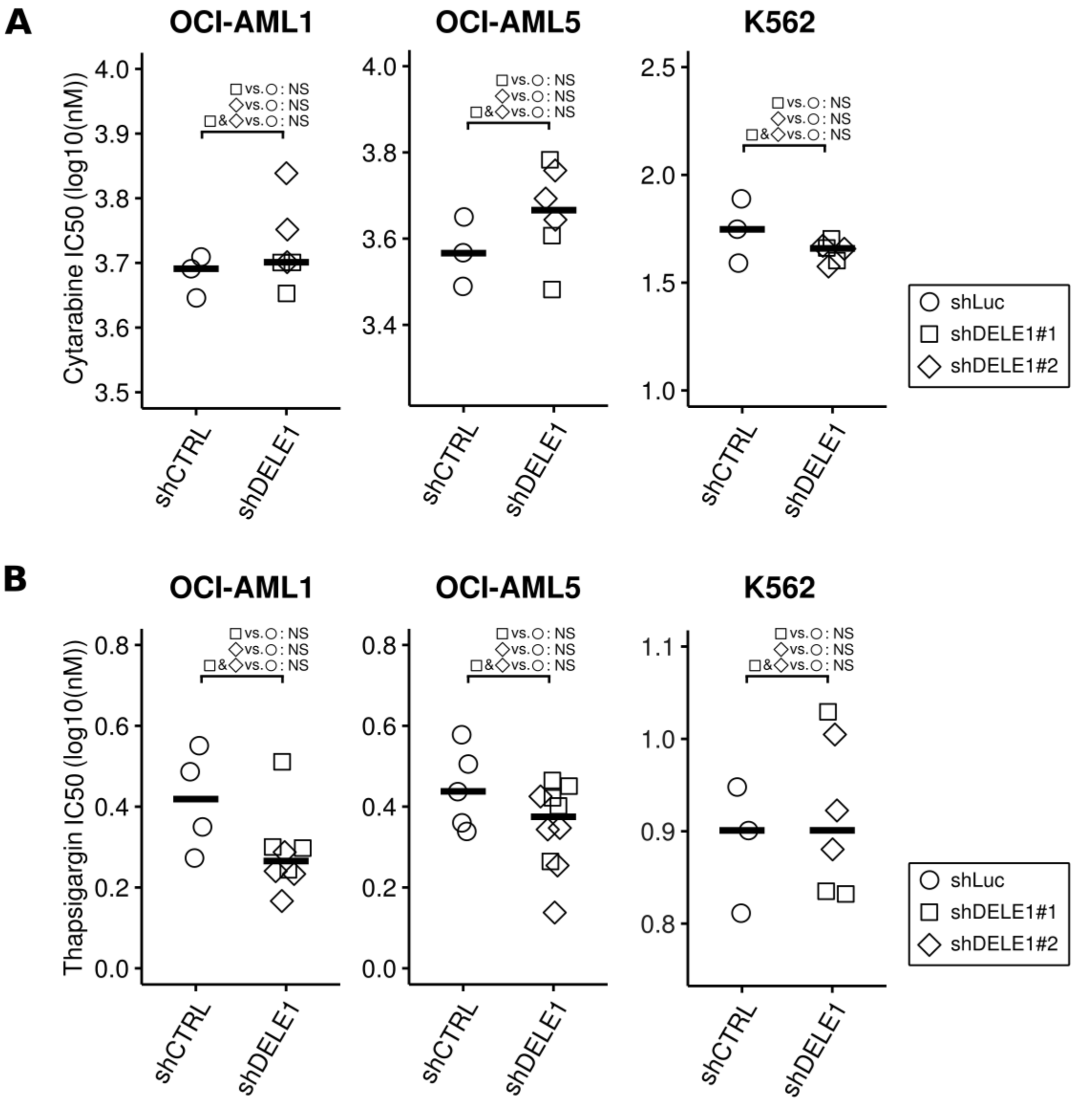

Figure S6. Cytarabine (**A**) and thapsigargin (**B**) IC50 values (log10 of concentrations in nM of the compound that inhibited cell growth by 50%) for OCI-AML-1, OCI-AML-5 cells and K562 cells infected with shRNA vectors targeting *DELE1* (shDELE1#1 and shDELE1#2) or the luciferase as control (shLuc). P-values resulting from Mann-Whitney tests comparing shDELE1#1/shDELE1#2 vs. shCTRL conditions are directly indicated on the figure.

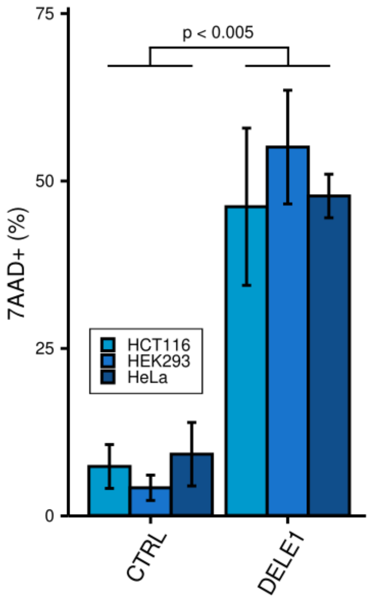

Figure S7. HCT116, HEK293T and HeLa cells were transiently transfected with empty (CTRL) or *DELE1*-encoding vectors (n=2 for each cell line, mean value + standard deviation). The P-value resulting from a Mann-Whitney test comparing CTRL and *DELE1* overexpression is directly indicated on the figure.

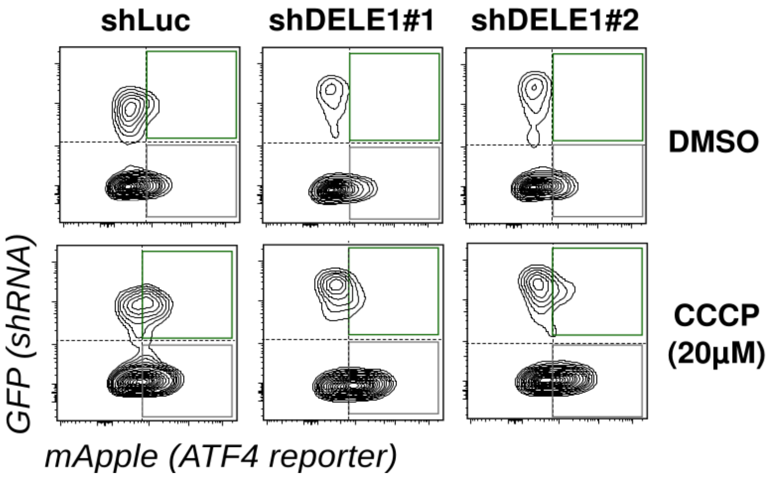

Figure S8. Representative FACS profile of GFP (shRNA) and mApple (ATF4 activity) expression in HL60 cells expressing shLuc (control), shDELE1 (shDELE1#1 and shDELE1#2) and exposed to CCCP (20 µM) for 24 hours.

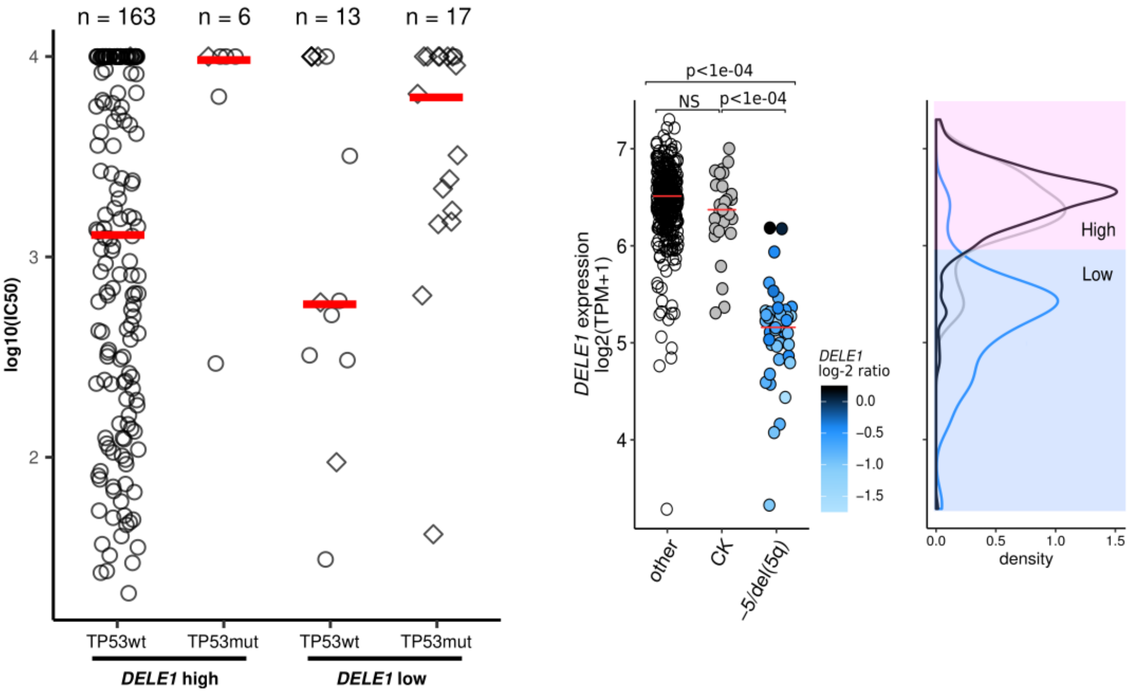

Figure S9. IC50 values (log10(nM)) resulting from Venetoclax treatment of primary AML specimens according to *TP53* mutational status and *DELE1* expression (left panel). *DELE1* “low” and “high” subgroups were defined according to expression distribution as represented in the right panel. These data were obtained from the previously published Leucegene study on the genetic characterization of ABT-199 sensitivity in human AML[30] (n = 199) and include 24 -5/del(5q) specimens used in the current study (depicted as diamonds in the left panel). Median values are indicated by red lines.

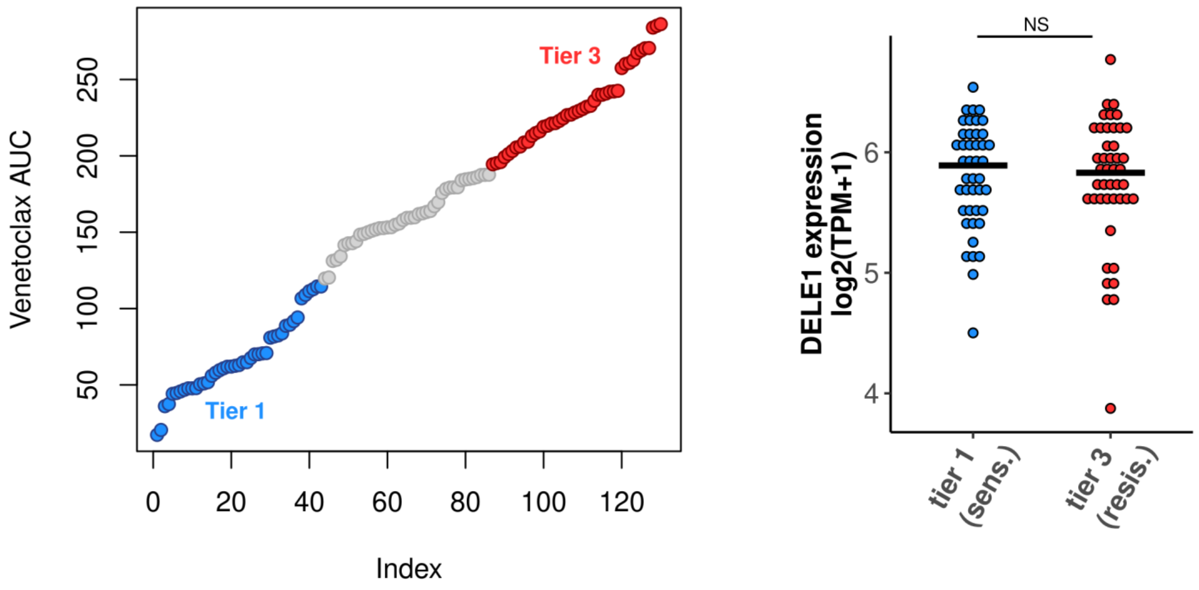

Figure S10. Distribution in ascending order of AUC values resulting from Venetoclax treatment of 130 AML specimens from the BEAT AML cohort (**left panel**) and dotplot representation of *DELE1* expression in AML classified in tiers 1 (sensitive, n = 43) and 3 (resistant, n = 44) according to drug sensitivity (**right panel**). Tiers 1 and 3 are depicted in blue and red, respectively.
